# Effects of Transcranial Direct Current Stimulation on Excitatory/Inhibitory Balance and Behavior in Children With Autism—A Randomized Controlled Study

**DOI:** 10.31083/AP46111

**Published:** 2025-08-28

**Authors:** Jiannan Kang, Yuqi Li, Wenqin Mao, Juanmei Wu, Xiaoli Li

**Affiliations:** ^1^Child Rehabilitation Division, Ningbo Rehabilitation Hospital, 315040 Ningbo, Zhejiang, China; ^2^State Key Laboratory of Cognitive Neuroscience and Learning, Beijing Normal University, 100875 Beijing, China

**Keywords:** autism spectrum disorder, brain modulation, electroencephalography, excitatory/inhibitory balance, transcranial direct current stimulation

## Abstract

**Background:**

Autism spectrum disorder (ASD) is a multifaceted neurodevelopmental disorder marked by impaired interactions and restricted interests, the pathophysiology of which is not fully understood. The current study explored the potential therapeutic effects of transcranial direct current stimulation (tDCS) on the neurophysiological aspects of ASD, specifically focusing on the brain’s excitatory/inhibitory (E/I) balance and behavioral outcomes, providing scientific guidance for ASD intervention.

**Methods:**

Forty-two children with ASD were randomly divided into either an active tDCS or sham tDCS group. Electroencephalography (EEG) recordings were conducted before and after stimulation to assess E/I changesusing EEG markers including α oscillations and the aperiodic exponent, and average spatial phase synchronization (ASPS) analysis and detrended fluctuation analysis (DFA) were performed. Behavioral changes were evaluated using the Autism Behavior Checklist (ABC) and the Social Responsiveness Scale (SRS).

**Results:**

Active tDCS resulted in significant increases in α oscillation power, reductions in α bandwidth, and improvements in γ-band ASPS and DFA values. Furthermore, participants in the active tDCS group exhibited improvements in behavioral scores on the ABC and SRS, with enhancements in social communication, sensory processing, and adaptive behavior. We found no significant changes in the sham group.

**Conclusion:**

These findings suggest that tDCS intervention effectively reduced brain excitability and improved E/I balance and behavioral outcomes in children with ASD. The results warrant further investigation into the efficacy and underlying mechanisms of tDCS for ASD treatment.

**Clinical Trial Registration:**

No: ChiCTR2400092790, https://www.chictr.org.cn/showproj.html?proj=249950.

## Main Points

1. Transcranial direct current stimulation (tDCS) can effectively regulate the brain excitability of children with autism spectrum disorder (ASD) and optimize their excitation/inhibition balance (E/I balance).

2. After tDCS intervention, children with ASD demonstrated a remarkable decline in behavioral scale scores, which evidently indicated an improvement in their behavioral performance.

3. Electroencephalography (EEG) markers were capable of effectively mirroring the alterations in the E/I balance and can be utilized to assess the efficacy of tDCS.

## 1. Introduction

Autism spectrum disorder (ASD) is a multifaceted neurodevelopmental condition marked by deficits in communication, repetitive behaviors and limited interests, typically manifesting in early childhood [1]. The etiology of this disorder is multifactorial, involving a complex interplay of genetics and environment [2]. Despite the increasing prevalence of ASD, its pathogenesis remains elusive. Currently, interventions for ASD children mainly involve traditional rehabilitation therapies designed to modify psychological states and correct abnormal behaviors through psychological and behavioral approaches. However, targeted therapies for ASD are actively being researched [3].

Traditional rehabilitative therapies for ASD include behavioral analysis, occupational therapy, and speech therapy. These methods are often time-consuming and have limited efficacy, placing significant burdens on families and caregivers [4]. Therefore, exploring new targeted treatments for ASD is of great importance. As neuromodulation technologies develop, transcranial direct current stimulation (tDCS) has emerged as a bright non-invasive neuromodulatory tool [5]. tDCS is a safe, inexpensive, non-invasive technique using a low DC voltage to brain’s cortex through electrodes placed on the scalp, thereby modulating brain excitability [6, 7]. Increasing evidence suggests that when tDCS is applied to the frontal regions, attention, learning, memory, alertness, and neurotransmitter levels can be significantly modulated [8, 9, 10, 11]. These effects make tDCS a hopeful tool for the treatment of ASD.

One of the most widely targeted areas for tDCS intervention was the dorsolateral prefrontal cortex (DLPFC), a critical region for executive functions that plays a crucial role in cognitive control, learning, planning, attention, and motivation [12, 13, 14]. However, research on the effects of tDCS in ASD has largely focused on behavioral assessments, which show positive effects on cognitive, social communication, and speech skills, but research on the direct effects of tDCS on the brains of children with ASD remains limited.

Study results have indicated that the brain’s excitatory/inhibitory (E/I) balance is crucial for understanding the pathophysiology of ASD [15]. Rubenstein and colleagues [16] identified a close relationship between changes in neuronal E/I balance and ASD, a finding that has been confirmed by several genetic studies. E/I balance is an intrinsic measure of excitability that does not depend on external stimuli [17]. A study has indicated that certain forms of ASD might be associated with an elevated E/I ratio, potentially specific to certain brain regions, reflecting local E/I imbalances and homeostatic regulation [18]. Other evidence suggests that the global deficits associated with ASD can also be described as neuronal hyperexcitability or hypo-excitability, emphasizing the importance of E/I balance [19].

Measuring E/I balance presents challenges, both directly and indirectly. Techniques like positron emission tomography (PET), magnetic resonance spectroscopy (MRS), and transcranial magnetic stimulation (TMS) are useful indicators of receptor occupancy and neurotransmitter activity, but they are limited by poor temporal resolution and high cost [19, 20]. On the other hand, electroencephalography (EEG) can capture real-time dynamics influenced by E/I fluctuations. High-density EEG systems also address spatial-resolution issues, allowing for precise source localization [21]. To assess E/I function, EEG markers are needed to capture both global and local E/I dynamics. Several methods, such as corrected α power, average spatial phase synchronization (ASPS), and detrended fluctuation analysis (DFA), have been developed to estimate the E/I balance [22, 23, 24, 25]. Spectral analysis of resting-state activity includes both periodic and non-periodic components. Periodic components reflect brain rhythms and their relationship to E/I balance [26]. Aperiodic components indicate brain excitability and E/I ratio [23]. ASPS in the γ band correlates strongly with cortical excitability [27], whereas DFA reveals long-term E/I correlations, with higher values indicating higher E/I balance [28].

In conclusion, this study was designed to use EEG surrogate markers to capture the E/I change in the resting-state brain of ASD children after receiving tDCS stimulation on the left DLPFC. Additionally, we combined behavioral assessments to provide a more comprehensive insight into the therapeutic potential of tDCS in modulating the neurophysiological basis of ASD.

## 2. Methods

### 2.1 Participants

Forty-two ASD children were divided into two groups at random, each with 21 participants: one group received active tDCS stimulation, and the other group received sham stimulation. Basic information about the participants is shown in Table 1. All participants were diagnosed by professional doctors based on DSM-5 criteria [29]. Written informed consent was obtained for all participants or their legal guardians. Their parents were thoroughly briefed on the entire experimental procedure before participation. This study was conducted in accordance with the Declaration of Helsinki and was approved by the Ethics Committee of Ningbo Rehabilitation Hospital (Approval Number: 2023006; Date: 24 March 2023).

**Table 1.** **Information of all participants**.

Group	Number of children	Male/female	Age ± SD (Years)
Real stimulation	21	13/8	5.4 ± 1.2
Sham stimulation	21	13/8	5.9 ± 1.2

SD, standard deviation.

Inclusion criteria for ASD children were: (1) assessed by a professional child psychiatrist and diagnosed with autism; (2) age 4–6 years; (3) written informed consent was obtained. Exclusion criteria were: (1) children with neurological disease history (e.g., epilepsy, brain injury, and brain surgery); (2) children receiving any medications; (3) previous exposure to tDCS, transcranial magnetic stimulation, or neurofeedback.

### 2.2 EEG Acquisition and Preprocessing

EEG was recorded in a quiet room. Each child sat comfortably in a chair, wearing an EEG cap, and keeping their eyes open. Typically, a guardian and a specialist monitored the condition of the child and make sure that the data were of good quality, with the specialist gently reminding the child to minimize blinking. EEG recordings lasted approximately 5–10 minutes. EEG data were recorded using a 128-channel HydroCel Geodesic Sensor Net (GSN) system (Electrical Geodesics, Inc. [EGI], Eugene, OR, USA). Throughout the EEG collection, electrode impedance was maintained below 50 kΩ. The sampling rate was set at 1000 Hz, with Cz as the reference electrode. Each child underwent EEG recording both before and after the tDCS intervention, resulting in two EEG recordings per participant.

Offline data analysis was performed using MATLAB R2016a (Version 9.0.0.341360, MathWorks Inc., Natick, MA, USA) [30] and EEGLAB (Version 13.5.4b, SCCN/University of California San Diego, La Jolla, CA, USA) [31]. To increase the speed of operation, we first down-sampled the EEG data to 200 Hz and filtered the EEG signals using a 1–45 Hz bandpass filter according to research plan. Independent component analysis was applied to remove eye blinks, muscle artifacts, and myoelectric components from the collected EEG signals [32]. Then, visual inspection of the data was performed to remove noisy segments. Finally, all the channels were re-referenced to the mean reference. We chose 19 electrodes for further analysis based on the 10–20 International Electrode System (Fp1, Fp2, F7, F3, Fz, F4, F8, T3, C3, Cz, C4, T4, T5, P3, Pz, P4, T6, O1, O2). This significantly decreased the complexity of data processing and reduced storage requirements. The 19 electrodes covered the brain regions we needed to analyze.

### 2.3 Stimulation Procedure

A direct-current stimulator (JX-tDCS-1, Huahengjingxing Medical Technology Co., Nanchang, Jiangxi, China) was applied to the scalp at a level of 1 mA. The impedance (7 × 4.5 cm) between the two saline-soaked electrodes was kept under 20 kΩ during stimulation. The anode was placed over the left DLPFC region, and the cathode was positioned above the right orbit. For active tDCS, the current was ramped up from 0 to 1 mA over 30 s, and then was ramped down from 1 mA to 0 over 20 min. For sham group, the electrode placement was identical to that of active tDCS, but the current was only applied to the participant’s scalp for the first 20 s.

### 2.4 Behavioral Evaluation

We recorded scores on the Autism Behavior Checklist (ABC) [33] and the Social Responsiveness Scale (SRS) [34] for children with ASD both before and after tDCS stimulation. These scales were completed by caregivers who were familiar with the ASD children, in order to screen for various behaviors.

The ABC includes 57 items divided into 5 subcategories: sensory; social relating; body and object use; language and communication skills; and social and adaptive skills. The SRS consists of 65 items categorized into 5 dimensions: social awareness; social cognition; social communication; social motivation; and autistic mannerisms. Generally, higher scores on these scales indicate more severe behavioral issues.

### 2.5 EEG Proxy Markers of E/I Estimate

(1) *Periodic and aperiodic components.* To analyze the periodic and aperiodic components of the power spectrum, the power spectrum density (PSD) of the 19 channels was calculated by the Welch method (2s Hamming window, 50% overlap). This approach allowed for modeling the power spectrum as a combination of periodic and aperiodic components. The PSD at each frequency point *f* is expressed as:

$$P=L+\sum_{n}G_{n}$$

Where L represents the aperiodic component of the power spectrum. This component typically described the overall trend in the power spectrum that followed a 1/f distribution or similar non-periodic scaling. $G_{n}$ was modeled as a single Gaussian function.

The aperiodic component L can be parameterized as:

$$L=b-\log_{10}\left(f^{x}\right)$$

In this parameterization, b represents the offset. X represents the exponent. The higher the exponent, the lower the E/I value.

The periodic component can be considered as a combination of multiple Gaussian functions $G_{n}$, with each individual Gaussian function represented as:

$$G_{n}=\alpha \times \exp\left[\frac{-{\left(f-C\right)}^{2}}{2\,\omega^{2}}\right]$$

Here, α represents the amplitude, ω represents the bandwidth of each peak, and C represents the center frequency.

The model-fitting was performed using the components described above, with the following settings: the peak width limits were set to 0.5–12 Hz, the range of fitting frequency was 1–40 Hz, aperiodic mode was set to ‘fixed’ with no inflection points. The periodic oscillatory component was obtained by subtracting the aperiodic component from the fitted spectrum. Final output of the algorithm included aperiodic parameters (exponent, offset) and periodic parameters (amplitude, bandwidth, center frequency).

(2) *Average spatial phase synchronization (ASPS). *This method has been demonstrated to be an effective indicator for monitoring E/I balance changes. It was computed across space rather than time and had been widely applied in neurology. In our study, we referred to commonly used frequencies for spatial phase synchrony and chose to calculate it in the γ band. We utilized Standardized Low-Resolution Brain Electromagnetic Tomography, Version 2002, The KEY Institute for Brain-Mind Research, University of Zurich, Zurich, Switzerland (sLORETA). to compute average spatial phase synchronization, creating regions of interest using 19 electrodes as seeds.

(3) *Detrended fluctuation analysis (DFA). *DFA was used to determine the self-similarity of signals. It calculated the detrended fluctuation of a signal, revealing its long-term correlations and nonlinear dynamic features. Increased DFA values indicated greater complexity and non-randomness in EEG signals. A previous study have shown that a higher DFA index reflected a greater E/I balance, with generally larger DFA indices corresponding to higher E/I ratios [28].

### 2.6 Statistical Analysis

Initially, Shapiro-Wilk test was performed to assess the normality of the data distribution for all variables. Since the aperiodic exponent, offset, periodic α oscillation power, bandwidth, DFA, and scores on the ABC and SRS scales, all followed a normal distribution, 2 × 2 repeated-measures analysis of variance (ANOVA) was used to test for significance of the parameters before and after stimulation. The main effects were Measurement Time (pre-tDCS, post-tDCS) and Group (active stimulation, sham stimulation). The results were corrected using the Greenhouse-Geisser correction. For parameters with significant interaction effects, post hoc examination was performed by SPSS 27.0.1 (Version 27.0.1, Release Build: 0082, IBM Corporation, Armonk, NY, USA). The significance level was set at *p* ≤ 0.05. For ASPS, the log-transformed data were analyzed by one-tailed *t*-statistics, with multiple comparisons corrected through a non-parametric permutation method involving 5000 randomizations. T-thresholds corresponding to a significance level of *p* ≤ 0.05 were calculated using statistical utilities from sLORETA.

## 3. Results

### 3.1 Aperiodic Exponent and Offset 

**Fig. 1.**
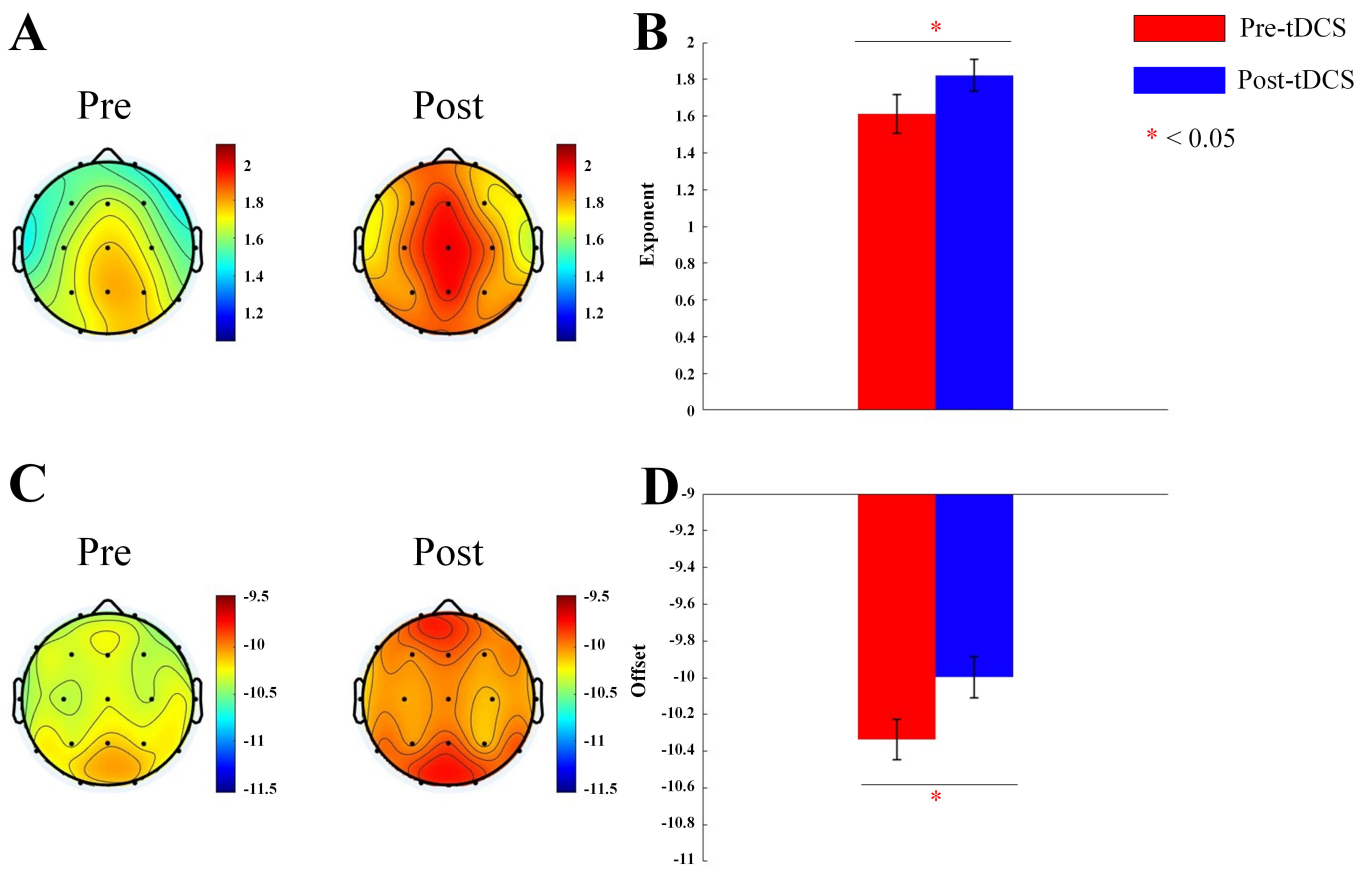
**Topographic map and bar graph results of aperiodic parameters**. (A) Topographic map of the exponent before and after active tDCS stimulation. (B) Bar graph of the exponent before and after active tDCS stimulation. (C) Topographic map of the offset before and after active tDCS stimulation. (D) Bar graph of the offset before and after active tDCS stimulation. tDCS, transcranial direct current stimulation. *, *p* < 0.05.

**Fig. 2.**
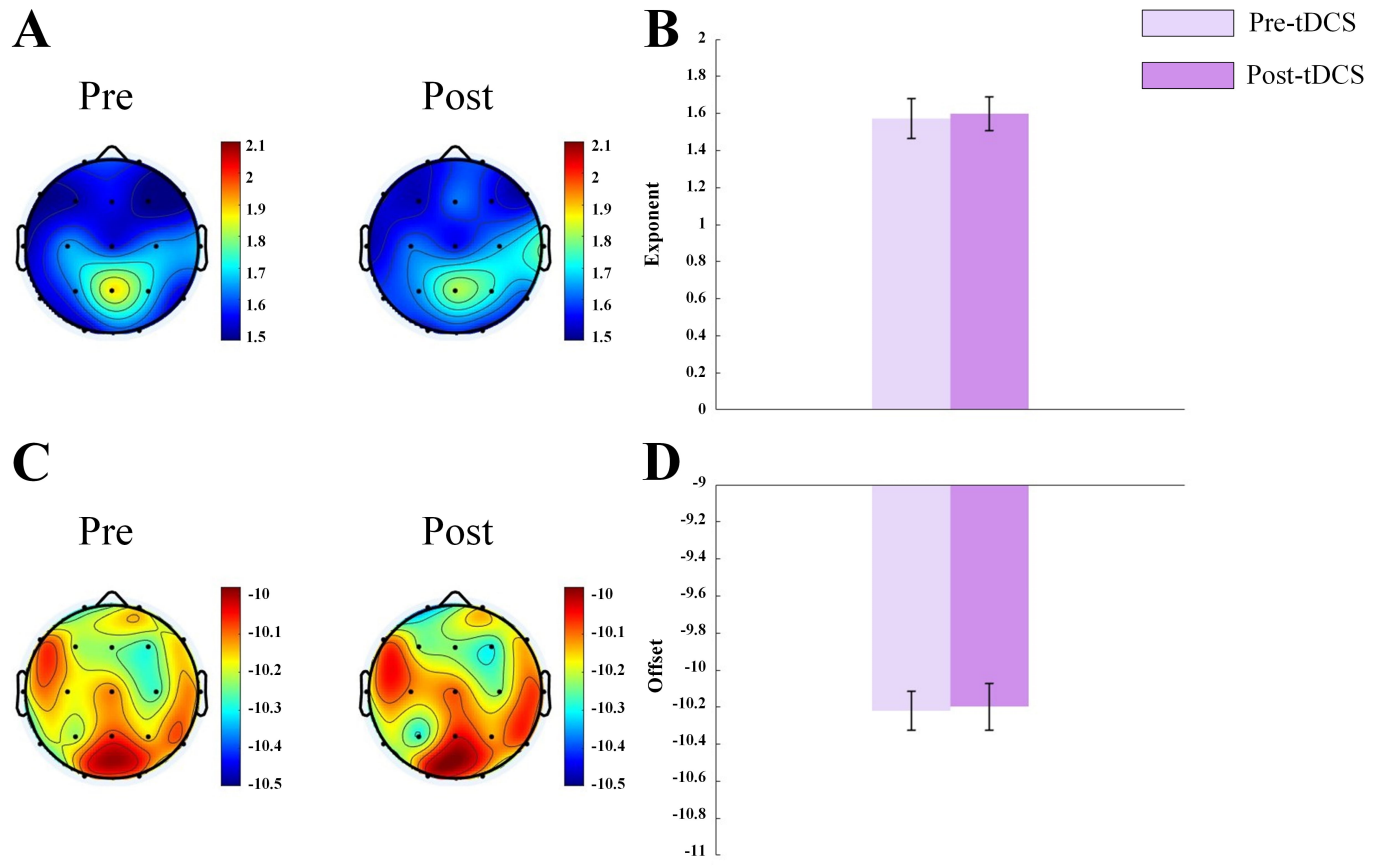
**Topographic map and bar graph results of aperiodic parameters**. (A) Topographic map of the exponent before and after sham tDCS stimulation. (B) Bar graph of the exponent before and after sham tDCS stimulation. (C) Topographic map of the offset before and after sham tDCS stimulation. (D) Bar graph of the offset before and after sham tDCS stimulation.

We calculated the aperiodic exponent and offset for 19 channels for active and sham tDCS stimulation groups. For the aperiodic exponent, Main effect of measurement time was significant (F = 131.390, *p* < 0.001). Main effect of group was significant (F = 18.451, *p* < 0.001). Interaction effect of Measurement Time × Group was significant (F = 79.583, *p* < 0.001). Post hoc test indicated the exponent increased significantly in active stimulation group. For the offset, Main effect of Measurement Time was significant (F = 142.015, *p* < 0.001). Main effect of Group was not significant (F = 1.509, *p* = 0.227). Interaction effect was significant (F = 108.772, *p* < 0.001). Post hoc examination indicated the offset increased significantly in active stimulation group (see Fig. 1). For ASD children receiving sham stimulation, no significant differences in the exponent or offset were observed (see Fig. 2). Our results showed that tDCS increased the aperiodic exponent and offset in the brains of children with ASD, demonstrating a regulatory effect on the E/I balance.

### 3.2 Corrected α Oscillations After Adjusting for Aperiodic Neural Activity

**Fig. 3.**
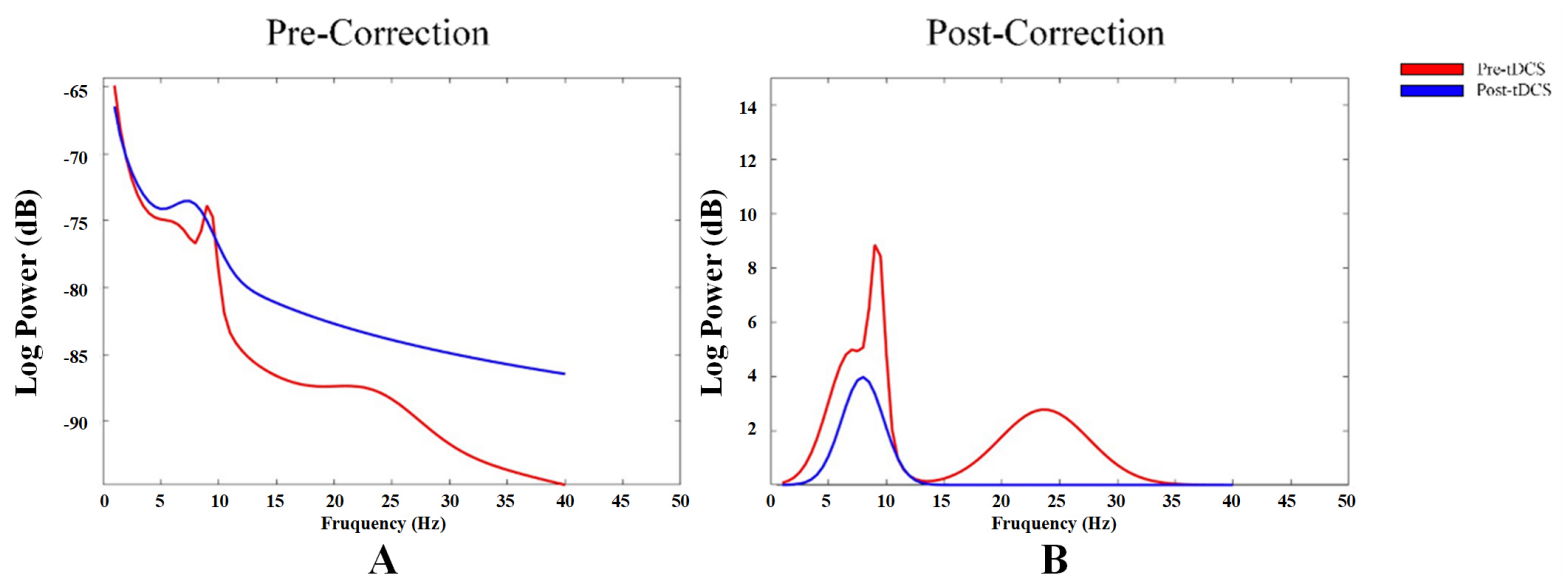
**Power spectrum changes in aperiodic neural activity before and after correction**. (A) Power spectrum before correction of aperiodic neural activity. (B) Power spectrum after correction of aperiodic neural activity.

By correcting for aperiodic activity and calculating the adjusted EEG power spectrum, we assessed the impact of the corrected 1/f-like aperiodic activity on power spectrum, as well as the characteristics of the corrected α oscillations. First, the power spectra from all channels across the entire brain were averaged. The power spectra before and after the correction of aperiodic activity were then compared (see Fig. 3).

**Fig. 4.**
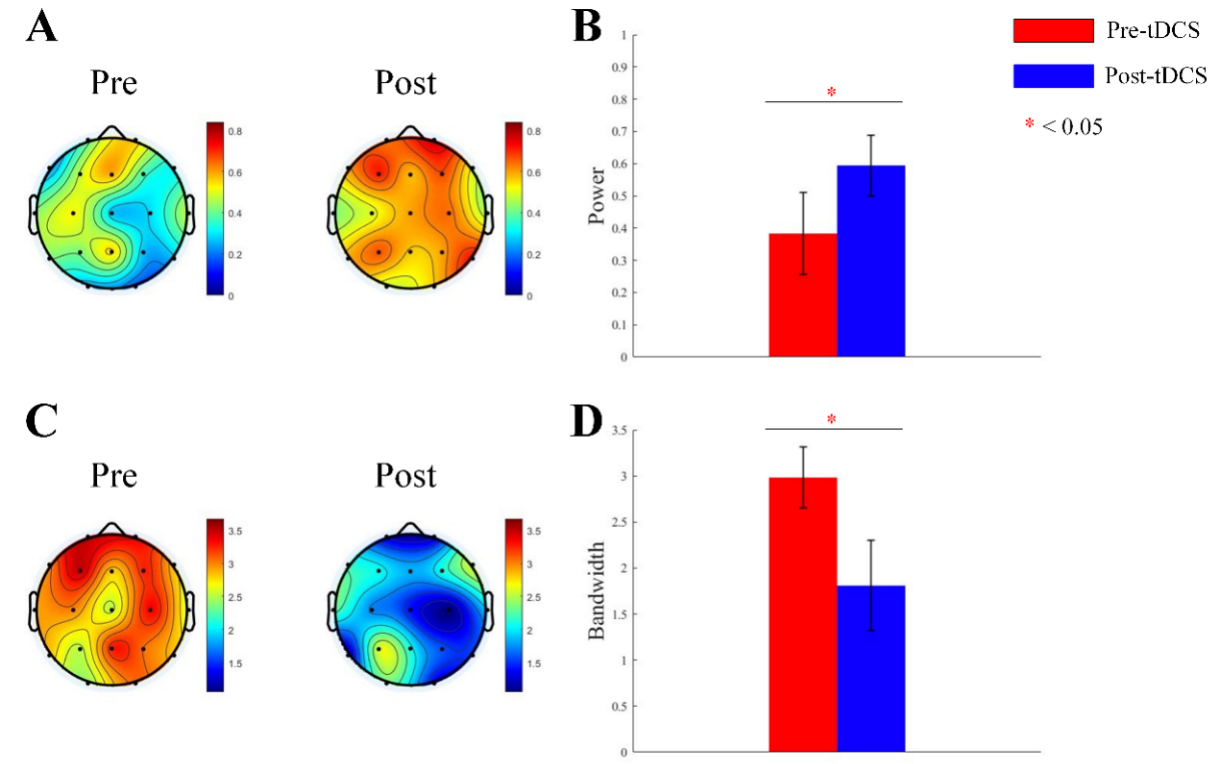
**Topographic map and bar graph results of periodic parameters**. (A) Topographic map of α oscillation power before and after active tDCS stimulation. (B) Bar graph of α oscillation power before and after active tDCS stimulation. (C) Topographic map of α oscillation bandwidth before and after active tDCS stimulation. (D) Bar graph of α oscillation bandwidth before and after active tDCS stimulation. *, *p* < 0.05.

**Fig. 5.**
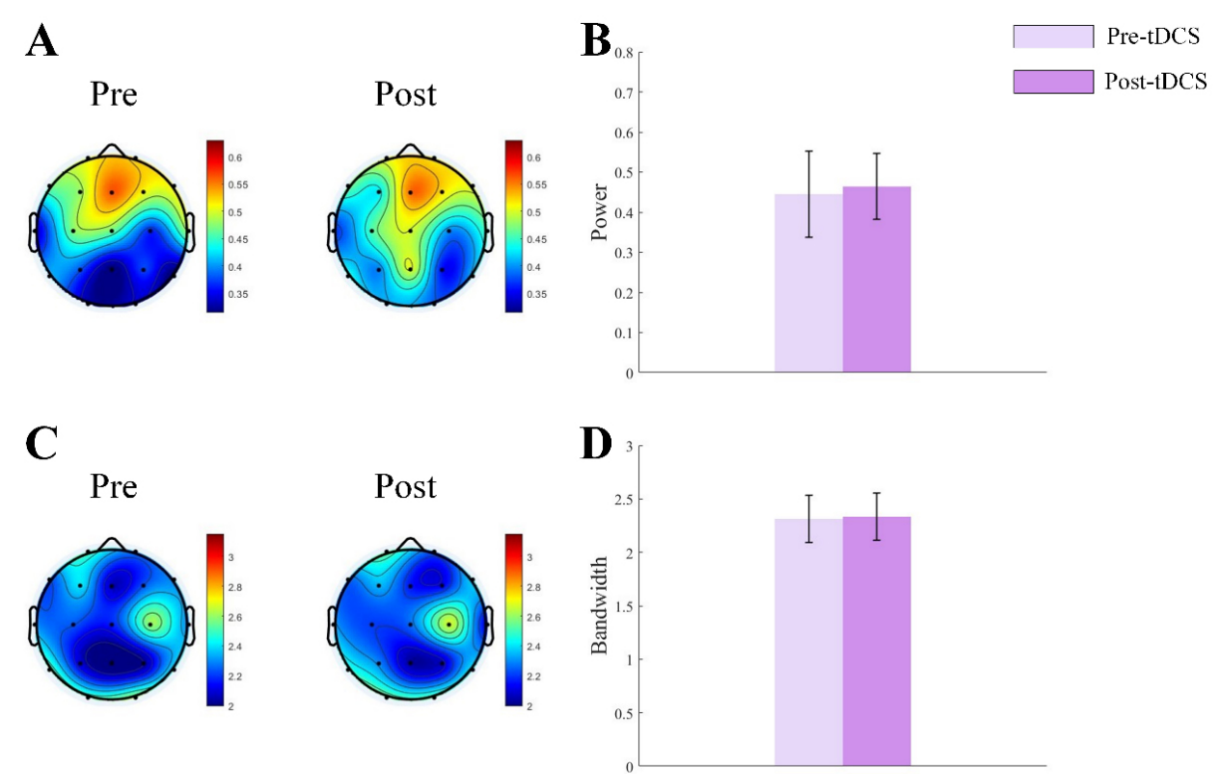
**Topographic map and bar graph results of periodic parameters**. (A) Topographic map of α oscillation power before and after sham tDCS stimulation. (B) Bar graph of α oscillation power before and after sham tDCS stimulation. (C) Topographic map of α oscillation bandwidth before and after sham tDCS stimulation. (D) Bar graph of α oscillation bandwidth before and after sham tDCS stimulation.

We obtained the periodic oscillatory components by subtracting the aperiodic components from the fitted spectrum. We analyzed power and bandwidth of α oscillations. For α power, Main effect of Measurement Time was significant (F = 36.877, *p* < 0.001). Main effect of Group was not significant (F = 1.485, *p* > 0.05). Interaction effect was significant (F = 25.751, *p* < 0.001). Post hoc examination indicated α power increased significantly in active stimulation group. For α bandwidth, Main effect of Measurement Time was significant (F = 51.452, *p* < 0.001). Main effect of Group was not significant (F = 1.018, *p* > 0.05). Interaction effect was significant (F = 55.084, *p* < 0.001). Post hoc examination indicated a significant decrease in α bandwidth for active stimulation group (see Fig. 4). For ASD children receiving sham stimulation, no significant differences in α oscillation power or bandwidth were observed (see Fig. 5). Our study found that tDCS stimulation increased the periodic α power and reduce the bandwidth in brains of ASD children, thereby suggesting a reduction in brain excitability and modulating the E/I balance.

### 3.3 Change in ASPS in the γ Band After Intervention

**Fig. 6.**
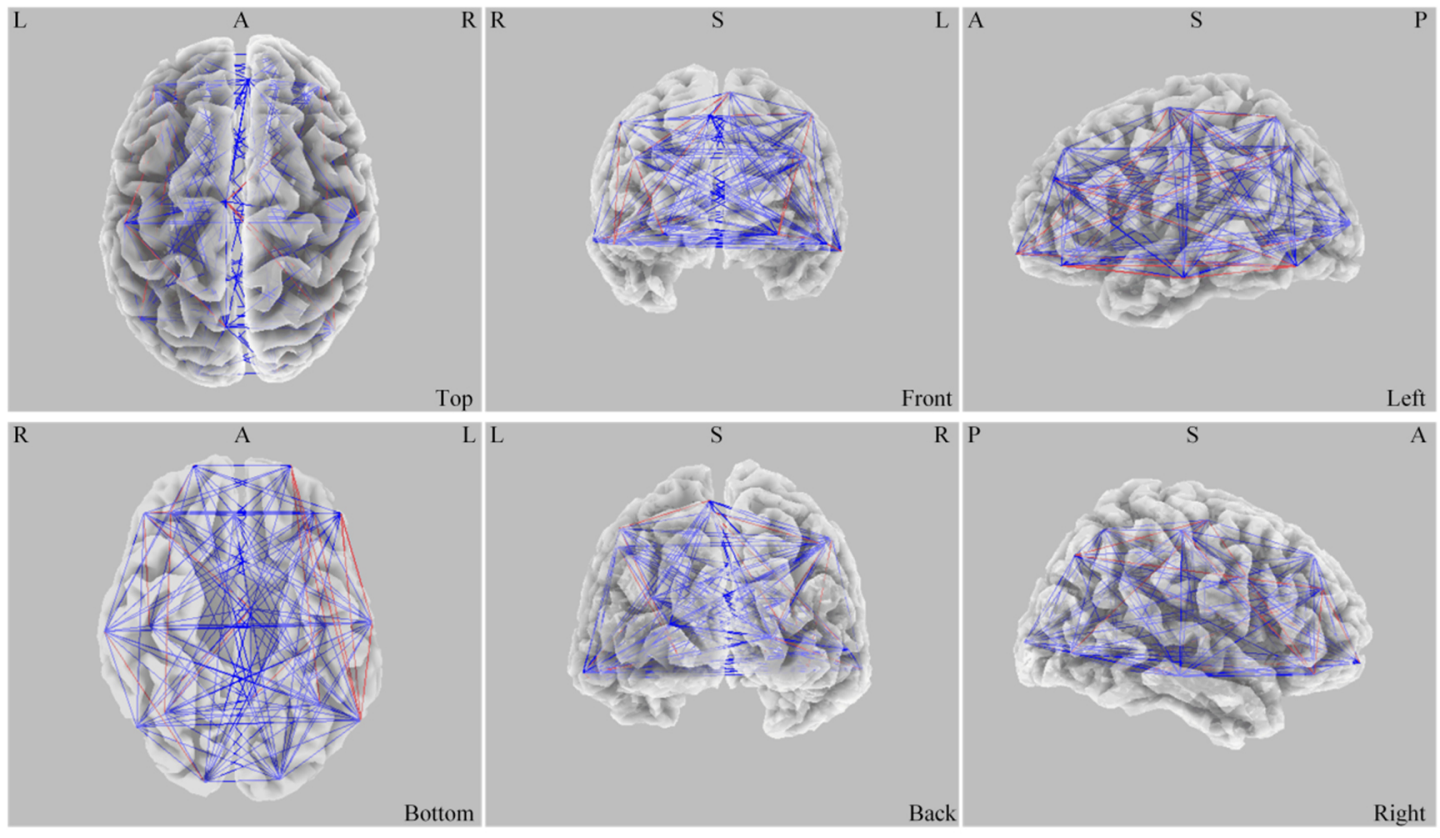
**Difference of ASPS before and after stimulation**. L, left; R, right; A, anterior; P, posterior; S, superior; ASPS, average spatial phase synchronization.

In the active group, ASPS in the γ band increased significantly in the brains of ASD children after stimulation (*p* < 0.05), as shown in Fig. 6. Blue lines in Fig. 6, indicating the ASPS in the brains of children with ASD, show that ASPS was significantly lower before the stimulation than after the stimulation. No significant difference was observed in the sham stimulation group. Our results showed that tDCS stimulation increased the ASPS in the γ frequency band in the brains of ASD children.

### 3.4 Change in DFA After tDCS Intervention

**Fig. 7.**
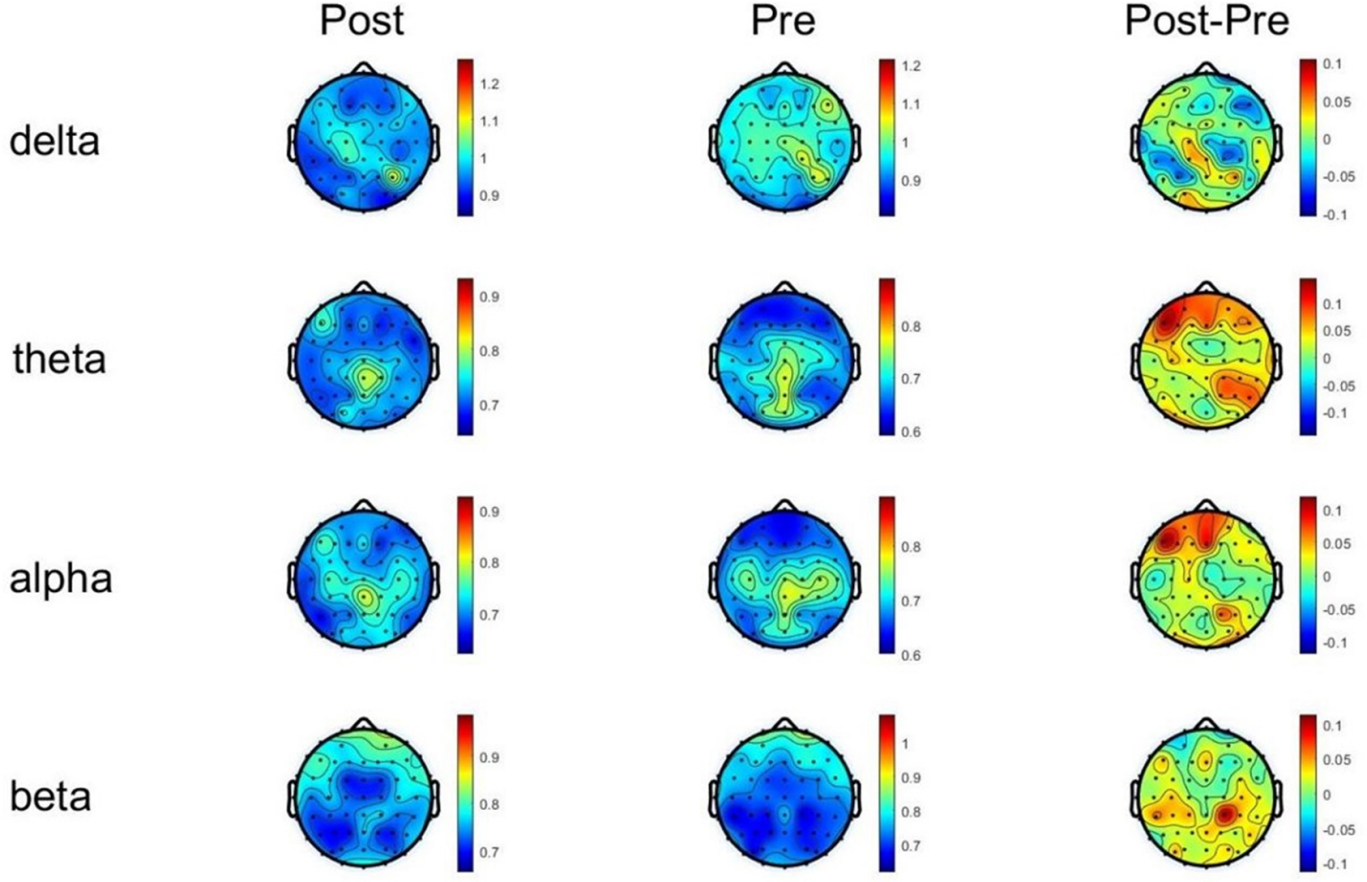
**DFA changes of each frequency band before and after tDCS stimulation**. DFA, detrended fluctuation analysis.

**Fig. 8.**
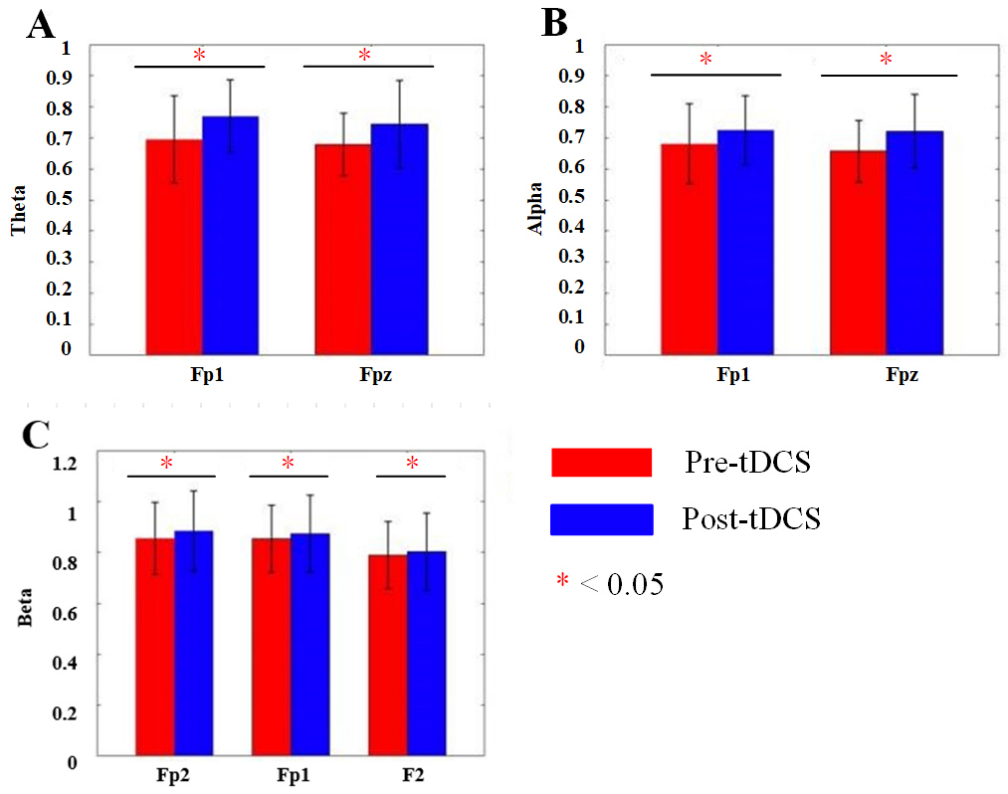
**Bar charts of DFA changes before and after tDCS in each frequency band**. (A) Bar chart of DFA values for the Fp1 and Fpz channels in the θ band before and after tDCS stimulation. (B) Bar chart of DFA values for the Fp1 and Fpz channels in the α band before and after tDCS stimulation. (C) Bar chart of DFA values for the Fp2, Fp1 and F2 channels in the β band before and after tDCS stimulation. *, *p* < 0.05.

In the θ frequency band, at the Fp1 channel, Main effect of Measurement Time was not significant (F < 1.0). Main effect of Group was not significant (F < 1.0). Interaction effect was significant (F = 14.751, *p* < 0.001). At the Fpz channel, Main effect of Measurement Time was not significant (F = 2.056, *p* > 0.05). Main effect of Group was not significant (F < 1.0). Interaction effect was significant (F = 9.242, *p* = 0.004). After post hoc examination, DFA in the θ band was found to have increased significantly in the active stimulation group. In the α frequency band, at the Fp1 channel, Main effect of Measurement Time was not significant (F < 1.0). Main effect of Group was not significant (F < 1.0). Interaction effect was significant (F = 12.226, *p* = 0.001). At the Fpz channel, Main effect of Measurement Time was not significant (F = 1.507, *p* = 0.227). Main effect of Group was not significant (F < 1.0). Interaction effect was significant (F = 19.529, *p* < 0.001). After post hoc examination, DFA in the α band was found to have increased significantly in the active stimulation group. In the β frequency band, at the Fp2 channel, Main effect of Measurement Time was not significant (F < 1.0). Main effect of Group was not significant (F < 1.0). Interaction effect was significant (F = 15.352, *p* < 0.001). At the Fp1 channel, Main effect of Measurement Time was not significant (F < 1.0). Main effect of Group was not significant (F <1.0). Interaction effect was significant (F = 14.244, *p* < 0.001). At the F2 channel, Main effect of Measurement Time was not significant (F < 1.0). Main effect of Group was significant (F = 7.592, *p* = 0.009). Interaction effect was significant (F = 13.470, *p* < 0.001). After post hoc examination, DFA in the β band was found to have increased significantly in the active stimulation group (see Figs. 7,8). For ASD children receiving sham stimulation, no significant differences in DFA were observed. Our results indicated that tDCS stimulation increased the DFA in the brains of children with ASD and reduced brain excitability.

### 3.5 Changes in Assessment Scores After tDCS Stimulation

We collected ABC and SRS scores of ASD children. In the ABC scale of active stimulation, for sensory scores, Main effect of Measurement Time was significant (F = 6.362, *p* = 0.023). Main effect of Group was significant (F = 15.201, *p* = 0.001). Interaction effect was significant (F = 16.602, *p* < 0.001). For social-relating scores, Main effect of Measurement Time was significant (F = 8.753, *p* = 0.009). Main effect of Group was not significant (F = 2.748, *p* = 0.117). Interaction effect was significant (F = 12.312, *p* = 0.003). For body- and object-use scores, Main effect of Measurement Time was significant (F = 16.488, *p* < 0.001). Main effect of Group was significant (F = 9.040, *p* = 0.008). Interaction effect was significant (F = 11.805, *p* = 0.003). For language- and communication-skills scores, Main effect of Measurement Time was significant (F = 14.286, *p* = 0.002). Main effect of Group was significant (F = 15.663, *p* = 0.001). Interaction effect was significant (F = 18.349, *p* < 0.001). Post hoc examination showed that scores in the 4 items above decreased significantly in the active stimulation group.

**Fig. 9.**
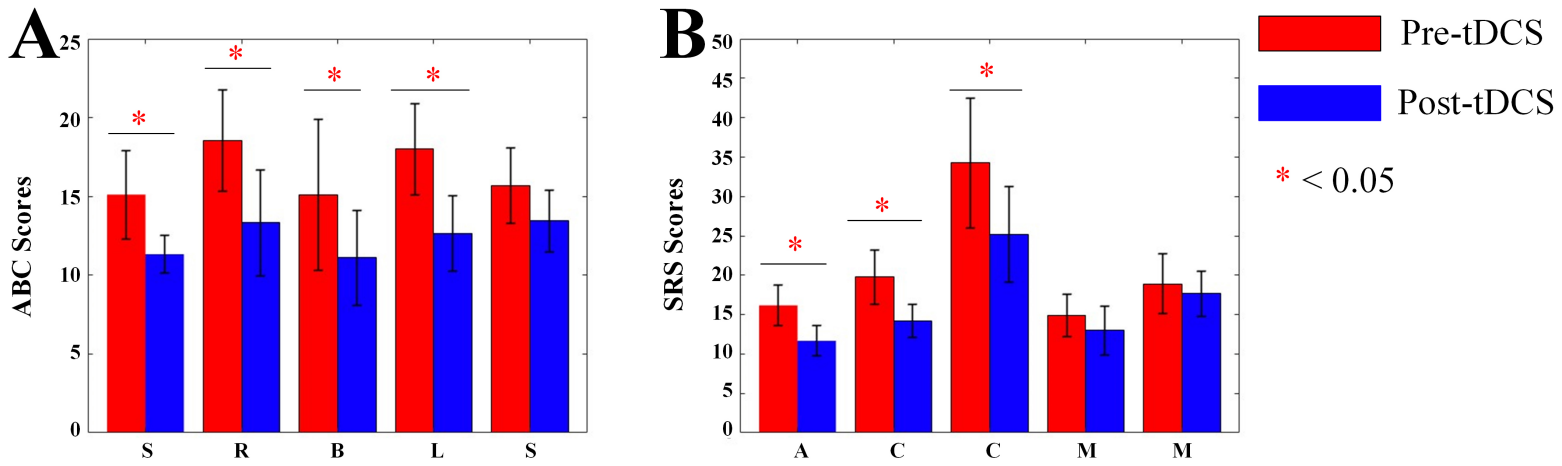
**Bar charts of ABC and SRS scores**. (A) Bar chart of ABC scale scores. (B) Bar chart of SRS scale scores. S, Sensory; R, Social relating; B, Body and object use; L, Language and communication skills; S (the second), Social and adaptive skills; A, Social awareness; C, Social cognition; C (the second), Social communication; M, Social motivation; M (the second), Autistic mannerisms; ABC, Autism Behavior Checklist; SRS, Social Responsiveness Scale. *, *p* < 0.05.

**Fig. 10.**
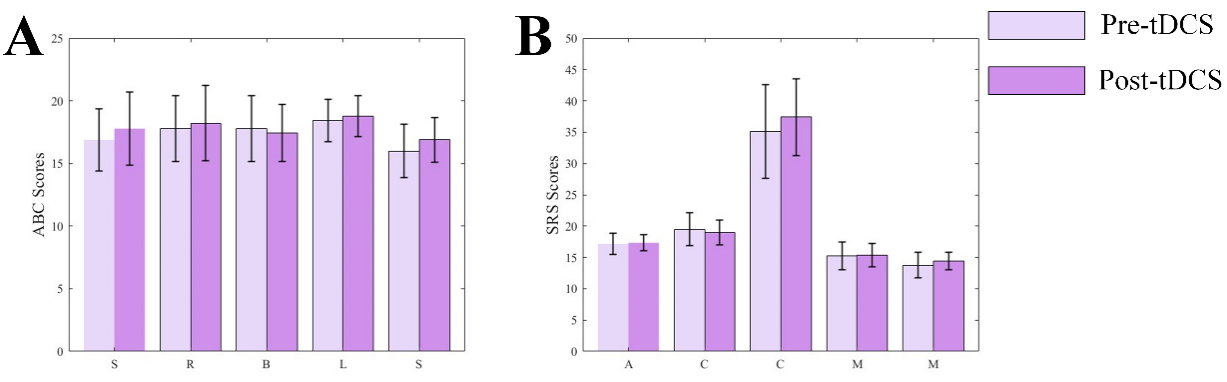
**Bar charts of ABC and SRS scores**. (A) Bar chart of ABC scale scores. (B) Bar chart of SRS scale scores.

In the SRS scale of active stimulation, for social-awareness scores, Main effect of Measurement Time was significant (F = 13.422, *p* = 0.001). Main effect of Group was significant (F = 38.428, *p* < 0.001). Interaction effect was significant (F = 15.567, *p* < 0.001). For social-cognition scores, Main effect of Measurement Time was significant (F = 22.213, *p* < 0.001). Main effect of Group was significant (F = 6.765, *p* = 0.016). Interaction effect was significant (F = 15.510, *p* < 0.001). For social-communication scores, Main effect of Measurement Time was significant (F = 15.598, *p* < 0.001). Main effect of Group was significant (F = 5.455, *p* = 0.029). Interaction was significant (F = 44.620, *p* < 0.001). Post hoc examination showed that scores in the 3 items above of ASD children significantly decreased in the active stimulation group (see Fig. 9). No significant differences were observed on the ABC or SRS scale scores for children receiving sham stimulation (see Fig. 10). Our study found that after tDCS stimulation, the behavioral-scale scores of children with ASD decreased, and their behavioral performance improved, demonstrating the positive effects of tDCS treatment for children with ASD.

## 4. Discussion

The current study explored the effects of tDCS on the E/I balance and behavioral symptoms in children with ASD. By using EEG-based surrogate markers of brain function, including aperiodic parameters, α oscillation power, bandwidth, ASPS, and DFA, we sought to understand how tDCS modulated neurophysiological activity and its potential therapeutic effects on ASD. Our results indicated that active tDCS targeting of the left DLPFC significantly modulated both the E/I balance and various behavioral indices in children with ASD, offering promising evidence for the therapeutic potential of neuromodulation in this population.

### 4.1 Modulation of E/I Balance by tDCS

One of the key findings in this study was the significant modulation of the aperiodic parameters of the EEG, which reflected the underlying excitability of the brain. Specifically, we observed that after active tDCS stimulation, the exponent and offset of the aperiodic component increased significantly, suggesting a shift toward a more balanced E/I ratio. This finding was consistent with previous research that has suggested that there is an elevated E/I ratio in individuals with ASD, reflecting neuronal hyperexcitability [16]. The increase in the aperiodic exponent and offset after active stimulation aligned with the notion that tDCS acts to normalize this imbalance, potentially reducing hyperexcitability and promoting neurophysiological homeostasis. These findings offered support for the hypothesis that neuromodulation through tDCS could be a valuable tool in restoring optimal brain excitability in individuals with ASD [35].

It is interesting to note that the sham stimulation group did not show any significant changes in the aperiodic exponent or offset, further validating the specificity of the effects of active tDCS. This result highlighted the potential of tDCS in directly modulating neural activity in a way that was not attributable to placebo effects. The absence of changes in the sham group suggested that the improvements observed in the active tDCS group were indeed related to the neuromodulatory effects of the stimulation, rather than to the general experience of participating in the study.

### 4.2 Changes in α Oscillations

The α oscillations, a key marker of brain rhythm, were associated with various cognitive functions, including attention, relaxation, and inhibitory control [36]. In the present study, we found that active tDCS significantly increased α power and decreased α bandwidth in children with ASD. This result suggested that tDCS enhances cortical inhibition in the brain, particularly in the regions targeted by the stimulation. The increased α power observed in the active tDCS group was consistent with previous research indicating that α oscillations were often linked to reduced cortical excitability and greater functional coordination across brain regions [26]. The reduction in α bandwidth further suggested improved synchronization within the brain’s neural network, possibly indicating a more efficient communication among brain regions, particularly in the frontal areas that are critical for executive functioning [37]. No significant changes in α oscillations were observed in the sham stimulation group, which again supported the notion that the observed effects in the active tDCS group were specifically due to the neuromodulatory impact of the stimulation.

### 4.3 Altered Spatial Phase Synchronization in the γ Band

Another important finding was the significant increase in ASPS in the γ frequency band after active tDCS. The γ band activity has been implicated in higher-order cognitive processes such as sensory integration, attention, and working memory, and it has been shown to be dysregulated in individuals with ASD [27, 38]. The increase in ASPS after tDCS suggested that the stimulation enhanced the synchronization of neural activity within the γfrequency band, potentially improving the coordination of brain regions involved in cognitive processing. This result was particularly relevant, as deficits in γ synchrony have been associated with the core symptoms of ASD, including difficulties with social communication and cognitive flexibility.

### 4.4 DFA and Long-term Correlations in Brain Activity

The application of DFA, to assess the long-term correlations and complexity of EEG signals, provided additional insights into the effects of tDCS on brain function [39]. In the present study, DFA values significantly increased in the θ, α, and β bands after active tDCS, indicating greater complexity and non-randomness in the brain’s neural signals. This finding suggested that tDCS promotes more adaptive and dynamic brain activity, potentially supporting better cognitive flexibility and behavioral regulation in children with ASD. The increase in DFA in multiple frequency bands supported the idea that tDCS enhances the overall neural organization of the brain, improving its ability to process and respond to external stimuli in a more coordinated manner. Again, no significant changes in DFA were observed in the sham group, reinforcing the notion that these neurophysiological improvements were specifically attributed to the active tDCS intervention.

### 4.5 Behavioral Improvements After Active tDCS

In addition to the neurophysiological changes, the study also assessed behavioral outcomes using the ABC and the SRS. Active tDCS led to significant improvements in various behavioral dimensions, including sensory processing, social relating, language and communication skills, and social awareness. These results aligned with a previous study that has showed the efficacy of tDCS in enhancing cognitive and social outcomes in ASD children [40]. The improvements in ABC and SRS scores, particularly in those that relate to social communication and social awareness, suggested that neurophysiological changes induced by tDCS may have translated into functional improvements in social interaction and communication skills—core areas of difficulty for individuals with ASD. The absence of significant changes in behavioral scores in the sham group further supported the effectiveness of the active tDCS intervention. These findings highlighted the potential of tDCS not only as a tool for modulating brain activity but also as a promising approach to address the behavioral symptoms of ASD.

### 4.6 Limitations and Future Directions

Although this study provided compelling evidence for the potential of tDCS in modulating neurophysiological activity and improving behavioral outcomes in children with ASD, there were several limitations that should be acknowledged. First, the sample size was small; future studies with larger cohorts are needed to confirm these findings and to further explore the generalizability of the results. Second, the long-term effects of tDCS remained unclear, and additional research is needed to investigate the sustainability of the observed improvements in both neurophysiological markers and behavioral symptoms. Finally, future studies should consider investigating other brain regions and stimulation parameters (e.g., current intensity, stimulation duration) to determine the optimal conditions for tDCS in the treatment of ASD.

## 5. Conclusion

The present study demonstrated that active tDCS applied to DLPFC significantly modulates E/I balance in the brains of ASD, leading to changes in neural synchrony and cortical excitability. These neurophysiological changes were associated with improvements in various behavioral domains, including social communication, sensory processing, and cognitive functioning. Given its non-invasive nature, low cost, and potential for enhancing brain function, tDCS holds promise as a therapeutic tool for children with ASD, offering a complementary approach to traditional behavioral interventions. It will also reduce the economic burden of ASD treatment on families and society. Further studies were needed to optimize the use of tDCS and explored its long-term impact on ASD symptomatology.

## Availability of Data and Materials

The data that support the findings of this study are available from the corresponding author upon reasonable request.
